# Comparison of the Effects of Perineural and Intraperitoneal Ozone Therapy on Nerve Healing in an Experimental Sciatic Nerve Injury Model

**DOI:** 10.3390/medicina60122097

**Published:** 2024-12-21

**Authors:** Burcu Ayık, Abdullah Ortadeveci, Fulya Bakılan, Dilek Burukoğlu Dönmez, Semih Öz, Cengiz Bal, Hilmi Özden, Onur Armağan

**Affiliations:** 1 Department of Physical Medicine and Rehabilitation, Faculty of Medicine, Eskişehir Osmangazi University, Eskişehir 26040, Turkey; fulyabakilan@gmail.com (F.B.);; 2 Department of Anatomy, Eskişehir Osmangazi University, Eskişehir 26040, Turkey; 3 Department of Histology and Embryology, Eskişehir Osmangazi University, Eskişehir 26040, Turkey; 4 Elderly Care, Health Care Services, Vocational School of Health Services, Eskişehir Osmangazi University, Eskişehir 26040, Turkey; 5 Department of Biostatistics, Eskişehir Osmangazi University, Eskişehir 26040, Turkey

**Keywords:** ozone, rat, sciatic nerve injury

## Abstract

*Background and Objectives:* The aim was to evaluate nerve healing using immunohistochemical, histological, and functional techniques and to compare the effects of two different therapeutic ozone application methods by perineural and intraperitoneal ozone treatment in rats with a crush injury model of sciatic nerve. *Materials and Methods:* Forty male Sprague Dawley rats were divided into four subgroups of ten rats each: (1) Control group: The left sciatic nerve incised and closed without crush injury, no treatment; (2) Paralyzed group: Crush injury to the left sciatic nerve, no treatment; (3) Perineural ozone group: Crush injury to the left sciatic nerve, treated with perineural ozone therapy; (4) Intraperitoneal ozone group: Crush injury to the left sciatic nerve, treated with intraperitoneal ozone therapy. The treatments were administered for a 14-day period. Hematoxylin and eosin (H&E) and toluidine blue staining were used for histological examination; TUNEL staining was used for immunohistochemical examination. Pinch test and rotarod performance assessment were utilized for functional evaluation. *Results:* The pinch test scores showed significant improvement in perineural and intraperitoneal ozone treatment groups after treatment (*p* < 0.001 and *p* = 0.003, respectively). The scores of myelin degeneration, vascular congestion, vascular wall thickness, inflammation, and toluidine blue and TUNEL staining were significantly lower in both ozone treatment groups compared to the paralyzed group (*p* < 0.001). Vascular wall thickness scores were significantly higher in the perineural ozone group compared to the control and intraperitoneal ozone groups (*p* = 0.004 and *p* = 0.013, respectively). The Schwann cell proliferation scores were significantly higher in the perineural ozone group compared to the control group and intraperitoneal ozone groups (*p* < 0.001). *Conclusions:* Both applications of ozone therapy accelerated the healing of nerve regeneration, reduced inflammation and apoptosis based on histopathological results, and enhanced nerve function in rats with sciatic nerve injury. Perineural ozone therapy has been demonstrated to be an efficacious alternative to systemic ozone treatments in the management of sciatic nerve injury. Further studies are needed to determine optimal ozone dosage and administration protocols for the treatment of nerve injury.

## 1. Introduction

Peripheral nerve injury is frequently caused by crush injuries, trauma, compression, occupational accidents, tumor excision, or iatrogenic causes. Following peripheral nerve injury, neuropathic pain, long-term disability, and a significant loss of the workforce are observed [1,2]. The development of structural and functional changes, including motor, sensory, and autonomic deficits, that occur as a consequence of peripheral nerve injury is attributed to oxidative stress [2,3,4]. Furthermore, oxidative stress may impair peripheral nerve regeneration [4,5].

Ozone gas (O_3_), which consists of three molecules of oxygen, is an oxidant substance. However, ozone at therapeutic doses stimulates the endogenous antioxidant systems by inducing mild oxidative stress. This stimulation provides the regulation of inflammation, immunomodulation, increased tissue oxygenation, suppression of infection, and an analgesic effect [6,7]. Therapeutic concentrations of medical ozone are accepted to be in the range of 5–60 µg/mL. This range is valid for local and systemic application techniques [8]. Medical ozone can be applied locally or parenterally [8,9]. It has been demonstrated by research that therapeutic ozone therapy can be employed safely at concentrations below 80 µg/mL [6,10,11].

In the literature, it has been established that therapeutic ozone applications have a positive impact on sciatic nerve healing in rat studies in which an experimental sciatic nerve cut or crush injury model was created. In these studies, ozone was applied locally via perineural injection or parenterally via intraperitoneal or rectal administration [7,11,12,13,14]. The administration of ozone to rats via intraperitoneal injections at varying doses and times demonstrated a reduction in the extent of myelin and axonal damage in models of sciatic nerve injury [7,11,13]. In a study in which a chronic constriction injury model was established in the rat sciatic nerve, a single peri-sciatic nerve ozone injection was found to reduce mechanical allodynia and thermal hyperalgesia [12]. However, no study comparing intraperitoneal and perineural ozone applications by creating a peripheral nerve injury model was found in the literature. Therefore, this study was designed to compare the contribution of two different ozone treatments to nerve regeneration in sciatic crush injury, a common clinical condition.

The primary aim of this study was to evaluate nerve healing using immunohistochemical, histological, and functional techniques in rats with a sciatic nerve crush injury model; the secondary aim was to compare the effects of two different methods of therapeutic ozone application by perineural and intraperitoneal ozone treatment at a concentration of 30 µg/mL.

## 2. Materials and Methods

This prospective, randomized, controlled study was conducted between November 2023 and January 2024 at Eskişehir Osmangazi University, Department of Physical Medicine and Rehabilitation. This study protocol was approved by the Local Ethics Committee for Animal Experimentation of the Eskişehir Osmangazi University (Decision number: 929-1) and supported by Eskisehir Osmangazi University Scientific Research Projects Committee (Project Code: TSA-2023-2701).

### 2.1. Animals

This study was carried out with 40 male Sprague Dawley rats (average weight: 300 g), who were kept in a controlled environment with humidity levels of 45–50% and temperature of 22 ± 2 °C, following a 12–14 h light–dark cycle. Animals were provided unrestricted access to food and water in a standard way. The animals were divided into 4 groups with 10 rats in each group, but 2 rats in the control group expired due to wound infection after surgery.

In group 1 (control), the left sciatic nerve trace was incised and closed at the skin level without creating a sciatic crush injury; so, no treatment was administered.

In group 2 (paralyzed), crush injury was created in the left sciatic nerve and no treatment was administered.

In group 3 (perineural), the left sciatic nerve crush injury was created and perineural ozone therapy was applied.

**Figure 1:**
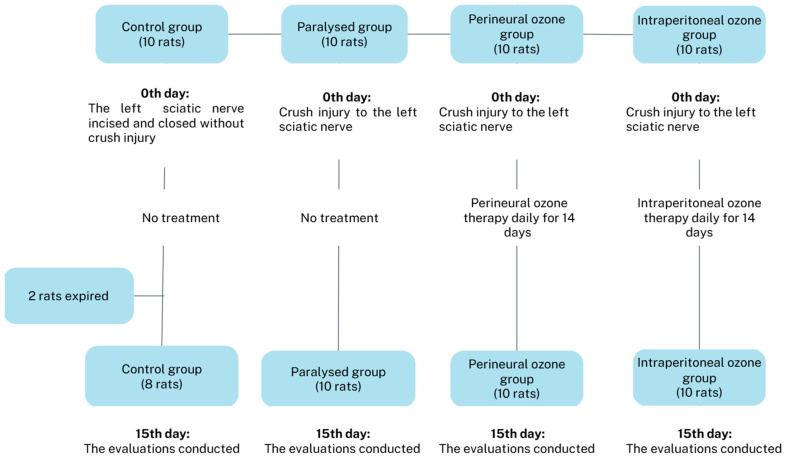
Study flow chart.

In group 4 (intraperitoneal), the left sciatic nerve crush injury was created and intraperitoneal ozone therapy was applied (Figure 1).

### 2.2. Surgery

**Figure 2:**
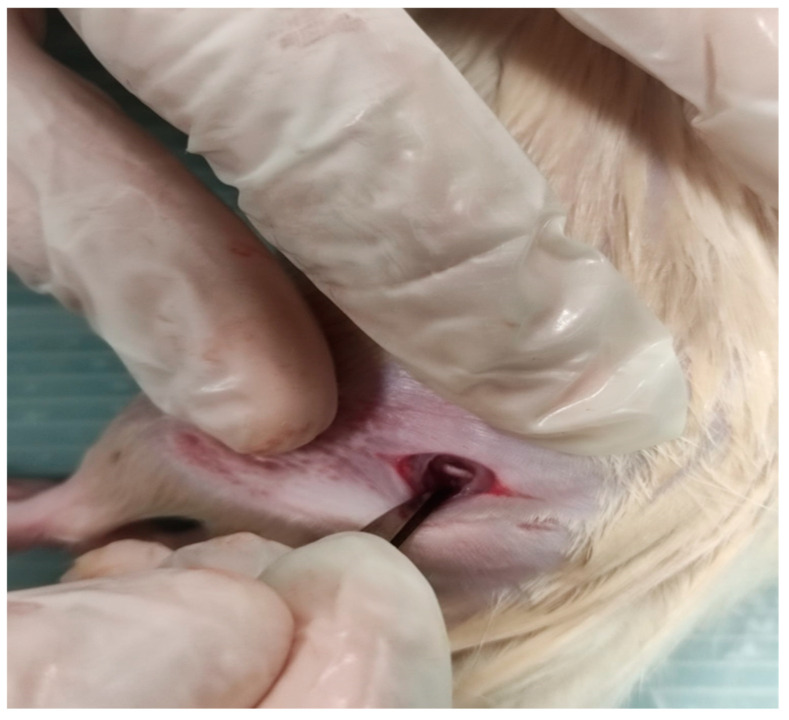
Region of the sciatic nerve dissection.

The surgical procedure was performed by clinical anatomists with at least 10 years of previous experience with experimental animals. The induction of deep anesthesia was accomplished through the intramuscular injection of 50 mg/kg ketamine (Ketalar^®^, Pfizer, İstanbul, Turkey) and 10 mg/kg xylazine (Rompun^®^, Bayer, İstanbul, Turkey). Following the adequate shaving and cleaning of the left posterior thigh, an oblique incision was made in the posterior aspect of the thigh by fixing the left posterior extremity and tail with the aid of a plaster. In the sciatic nerve injury groups, the left sciatic nerve was accessed between the vastus lateralis and biceps femoris muscles using scissors and forceps (Figure 2). The nerve was crushed with clamp and compressed for 90 s. In the control group, only a skin incision was made in the same area and sutured with a 2.0 suture material [11].

### 2.3. Ozone Treatment

Ozone was generated by a Medozon compact generator (Herrmann Apparatebau GmbH, Kleinwallstadt, Germany), with O_3_ concentration being monitored in real time using photometric measurement and validated through iodometric titration in accordance with the guidelines set by the International Ozone Association.

Both ozone application methods were applied once a day, every day for 14 days.

Perineural ozone injection: A volume of 1 mL and a concentration of 30 μg/mL ozone (O_3_) was injected into the lateral edge of the long head of the biceps femoris at the incision site with an ozone-resistant injector [6,12]. Ozone, injected through the incision by passing through the muscle and fascia level with a 22 G needle tip, shows perineural spread as it is a gaseous molecule.

The administration of intraperitoneal ozone: Ozone at a concentration of 30 μg/mL and a dose of 0.7 mg/kg was administered intraperitoneally into the left lower quadrant of the rats using an ozone-resistant syringe [14].

### 2.4. Functional Assessment

Pinch test: The sensory function of the rats was evaluated using a pinch test. The rats were gently held and pinched with a clamp without being exposed to excessive stress. The rats were graded according to the strength of their withdrawal reflex: no withdrawal reflex (grade 0), weak response (grade 1), moderate response (grade 2), and severe response (grade 3). The evaluations were performed on days 1 and 15.

Rotarod performance assessment: A rotarod with a diameter of 5 cm was used to evaluate sciatic nerve motor function. Animals were subjected to training sessions on the rotarod during 14-day injection periods. In these sessions, the animals were placed on the rods at 25 rpm, and the duration of their stay on the rod was recorded. Training sessions were conducted on two separate days, three times a day, with three-to-four-hour intervals. The evaluations were conducted on the 15th day following 14 sessions of treatment [15]. To assess the motor functions, incisions were made on the rats in the control group, revealing only the deficit due to nerve damage.

### 2.5. Microscopic Assessment

Histological and immunohistochemical analyses were performed by a histology specialist with 20 years of experience in the field. A two-millimeter segment of the sciatic nerve collected on the same side as the injury. Nerve samples in the control group were taken from a 2 mm segment in the region corresponding to the equivalent part of the lesioned nerve. The specimens were obtained 3 mm away from to the crush injury site, as in a similar study [16], following the sacrifice of the rats under deep anesthesia on the 15th day after 14 sessions of treatment. Nerve tissue samples from rats were placed in 10% formalin fixative for 48 h in order to prepare them for histologic examination under light microscopy. Subsequently, the cleaned nerve tissue samples were embedded in paraffin. Tissue sections of 5 micrometer thickness were extracted from the paraffin blocks using a microtome (Leica CM1900; Lyca Microsystems GmbH, Wetzlar, Germany). The taken tissue sections were deparaffinized and some of the sections were stained with hematoxylin and eosin (H&E) dye, some of which were stained with toluidine blue to be evaluated under a light microscope (Olympus BH-2, Tokyo, Japan). In addition, nerve sections obtained from all experimental groups were subjected to TUNEL staining followed by immunohistochemical examination at different magnifications, and light microscopy images were obtained. Images of all nerve specimens were captured using an Olympus DP-70 digital camera (Olympus, Tokyo, Japan). The photographed specimens were scored and statistically analyzed.

Grading according to histological and immunohistochemical staining was based on a score of 0–3 for each section [17].

H&E-stained specimens were scored for myelin degeneration, vascular congestion, vessel wall thickening, inflammation, and Schwann cell proliferation as: 0: absent, 1: minimally present, 2: moderately present, and 3: highly present.

The light microscopic examination of toluidine blue-stained specimens was conducted, scoring for mast cells, myelinated axon structures, and vascular formations. The following scores were adopted: 0: none, 1: minimal staining, 2: moderate staining, and 3: advanced staining.

The TUNEL method was applied to determine apoptotic evaluations in nerve tissue specimens. Sciatic nerves obtained from all experimental groups were subjected to TUNEL staining, followed by immunohistochemical examination at different magnifications and light microscopy images were obtained. All specimens were scored as: 0: none, 1: minimal staining, 2: moderate staining, and 3: advanced staining.

### 2.6. Statistical Analysis

The study sample size was determined as 8 rats in each group when calculated with the G*Power version 3.1.9.2 software (Heinrich Heine Universität Düsseldorf, Düsseldorf, Germany), with a 90% power and a 5% type-1 error rate using statistical information obtained from a pilot study [14]. The number of animals used was determined as 10 according to the resource equality method, similar to the literature [13,14].

IBM SPSS for Windows 21 (SPSS Inc., Chicago, IL, USA) was used to analyze the data. The Shapiro–Wilk test was used to determine the suitability of the variables for normal distribution. Nonparametric tests were used to compare the groups. The Kruskal–Wallis H test was used to compare independent groups according to distribution forms. A post hoc multiple comparisons test was used to determine different groups. Wilcoxon paired test and Friedman test were used to compare dependent groups. Median (25–75%) statistics for quantitative data were used to summarize the data. A *p*-value < 0.05 was considered statistically significant.

## 3. Results

A comparison of the pinch test scores between the groups before treatment revealed significantly lower scores in the paralyzed, perineural, and intraperitoneal groups compared to the control group (all groups: *p* < 0.001). After treatment, a statistically significant difference in the pinch test scores was observed between the study groups (*p* = 0.001). This difference was significantly higher in the control and ozone groups compared to the paralyzed group (control group *p* < 0.001; perineural and intraperitoneal groups: *p* = 0.005) (Table 1).

Within each group, the pre- and post-treatment comparison of the pinch test scores showed significant improvements in the perineural and intraperitoneal groups (perineural, *p* < 0.001; intraperitoneal, *p* = 0.003). However, there was no significant difference between the groups in the evaluation of the rotarod performance times after treatment (*p* = 0.402) (Table 1).

The statistical analysis revealed significant differences between groups in all histopathological parameters, including myelin degeneration, vascular congestion, vascular wall thickness, inflammatory cell infiltrate, and Schwann cell proliferation (*p* < 0.001). When comparing myelin degeneration scores between groups, it was observed that the scores of the control, perineural, and intraperitoneal groups were significantly lower than those of the paralyzed group (*p* < 0.001, *p* = 0.001, and *p* < 0.001, respectively). Additionally, the perineural ozone group had significantly higher scores compared to the control group (*p* = 0.048) (Table 2).

In terms of vascular congestion and inflammation scores, the control, perineural, and intraperitoneal groups had significantly lower scores compared to the paralyzed group (all groups: *p* < 0.001). Vascular wall thickness scores were significantly lower in the control, perineural, and intraperitoneal ozone groups compared to the paralyzed group (*p* < 0.001, *p* = 0.024, and *p* < 0.001, respectively). Moreover, vascular wall thickness scores were higher in the perineural ozone group compared to the control and intraperitoneal ozone groups (*p* = 0.004 and *p* = 0.013, respectively) (Table 2).

**Figure 3:**
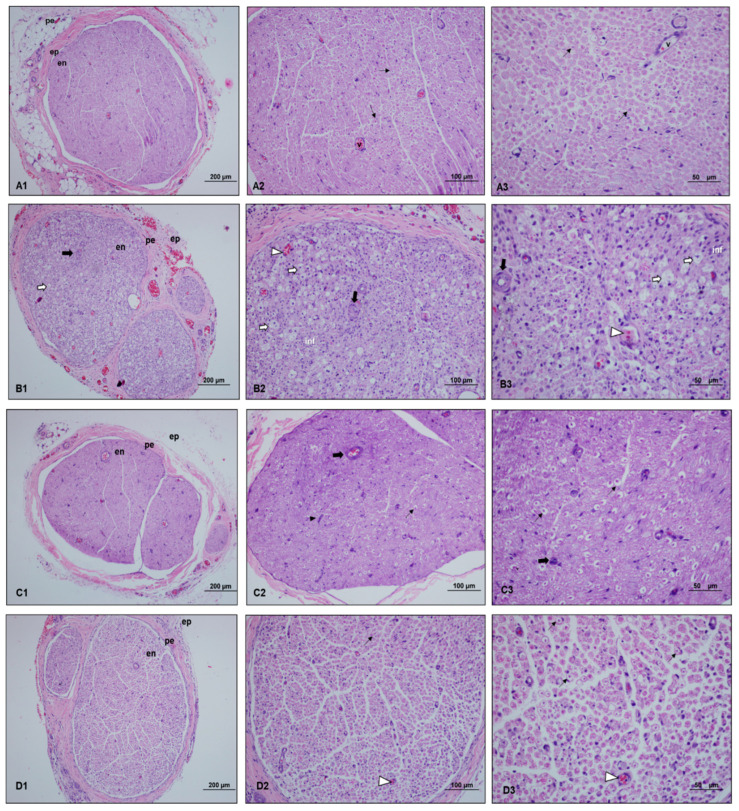
Light microscopy examination images of sciatic nerve specimens from each group. ((**A1**–**A3**): Control group, (**B1–B3**): Paralyzed group, (**C1**–**C3**): Perineural ozone group, (**D1**–**D3**): Intraperitoneal ozone group.) (ep: epineurium, pe: perineurium, en: endoneurium, inf: inflammation, v: vessel, Hematoxylin and eosin (H&E), scale bar: 200 µm-×10, 100 µm-×20, 50 µm-×40.) Myelinated axon structures (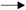), myelinated axon structures observed in degenerated vacuolar structure (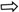), vascular congestion (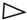), vascular wall thickening (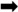), and proliferating Schwann cells (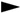). Light microscopy examination of the sciatic nerve specimens taken from the control group after hematoxylin–eosin staining showed a normal histological structure characterized by intact myelinated axons and vascular structures. In the paralyzed group, the light microscopy examination revealed degenerated vacuolar structures, vascular congestion, vascular wall thickening, and inflammation in myelinated axon structures. In the perineural ozone group, near normal myelinated axon structures as well as decreased vascular wall thickening and proliferated Schwann cells were observed in some areas. In the intraperitoneal ozone group, although a small number of vascular congestion was observed in some areas, it revealed a histologic structure close to normal in general evaluation.

Regarding Schwann cell proliferation, the control group scores were significantly lower (*p* = 0.002), while the perineural ozone group scores were higher (*p* = 0.018) compared to the paralyzed group. Furthermore, the perineural ozone group scores were significantly higher than the control group and the intraperitoneal ozone group (*p* < 0.001) (Table 2) (Figure 3).

**Figure 4:**
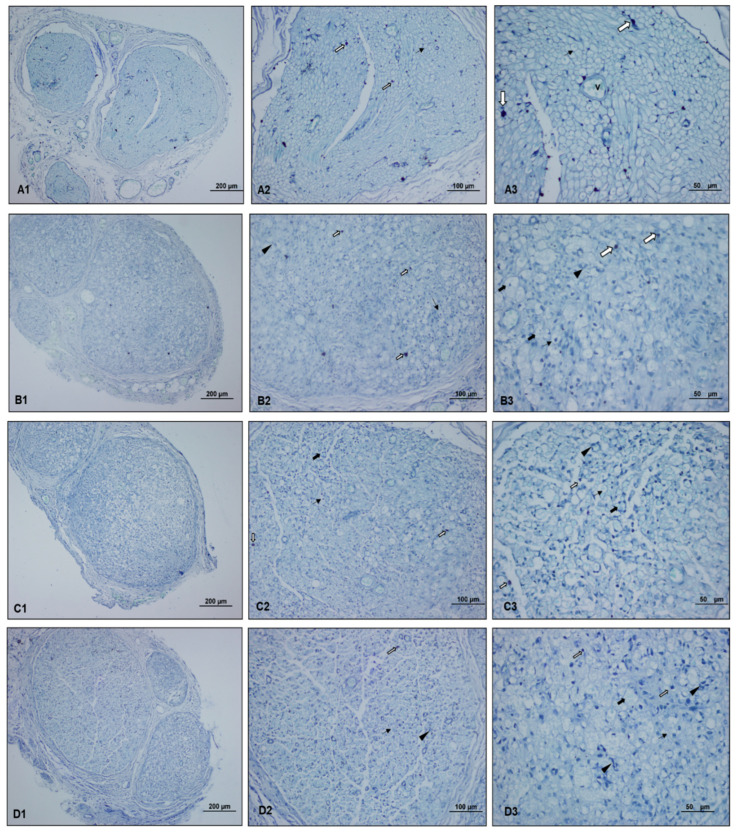
Light microscopy images of toluidine blue-stained preparations of sciatic nerve specimens from each group. ((**A1**–**A3**): Control group, (**B1**–**B3**): Paralyzed group, (**C1**–**C3**): Perineural ozone group, (**D1**–**D3**): Intraperitoneal ozone group) (Toluidine blue, scale bar: 200 µm-×10, 100 µm-×20, 50 µm-×40.) Mast cells (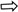), myelinated axon structures observed in degenerated vacuolar structure (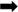), normal myelinated axon structures (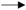), and proliferating Schwann cells (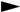). The light microscopy examination of toluidine blue-stained sciatic nerve specimens revealed a normal histological structure characterized by mast cells, myelinated axon structures, and vascular formations in the control group. In the paralyzed group, mast cells, degenerated vacuolar structures in myelinated axon structures, and few proliferating Schwann cells were detected. In the perineural and intraperitoneal ozone groups, mast cells and myelinated axon structures, reduced degenerated myelinated axon structures, and proliferating Schwann cells were detected in some areas.

The comparison of toluidine blue staining scores showed that the control (*p* < 0.001), perineural (*p* = 0.017), and intraperitoneal (*p* = 0.002) ozone groups had significantly lower scores than the paralyzed group. The control group scores were also significantly lower than the perineural ozone group scores (*p* = 0.011) (Table 2) (Figure 4).

**Figure 5:**
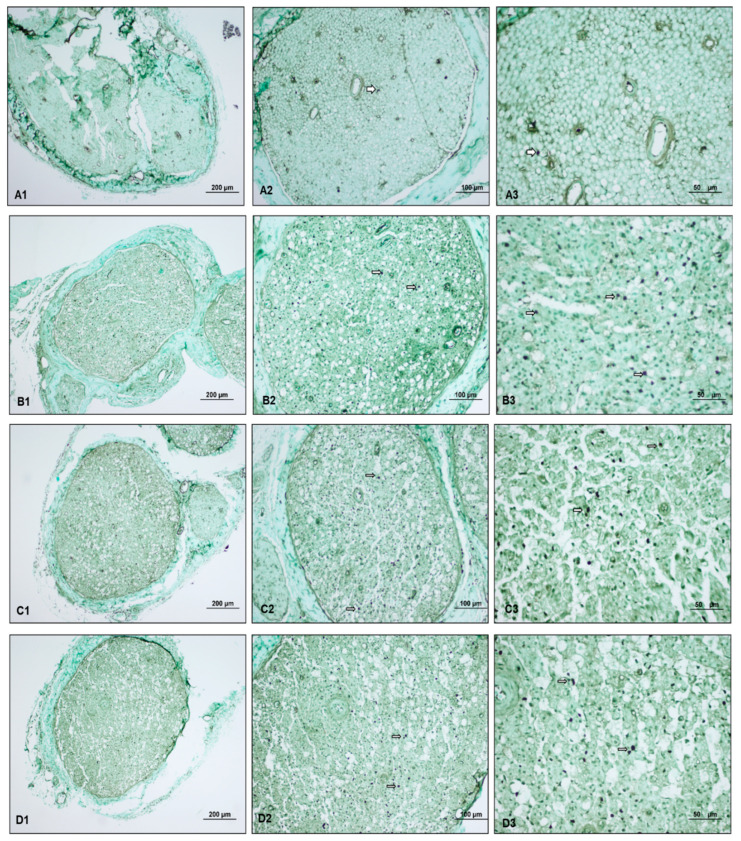
Immunohistochemical examinations following TUNEL staining of sciatic nerve specimens from each group. ((**A1**–**A3**): Control group, (**B1**–**B3**): Paralyzed group, (**C1**–**C3**): Perineural ozone group, (**D1**–**D3**): Intraperitoneal ozone group.) (TUNEL, scale bar: 200 µm-×10, 100 µm-×20, 50 µm-×40.) TUNEL staining (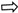). The light microscopy examination of the specimens subjected to TUNEL staining revealed minimal positive staining in the control group, advanced positive staining in the paralyzed group, and moderate positive staining in both ozone groups.

In terms of TUNEL test scores, the paralyzed (*p* < 0.001), perineural (*p* = 0.003), and intraperitoneal (*p* = 0.022) ozone groups had significantly higher scores compared to the control group. The scores of the perineural and intraperitoneal ozone groups were significantly lower than the paralyzed group (*p* = 0.012 and *p* = 0.001, respectively) (Table 2) (Figure 5).

## 4. Discussion

The present study was designed to assess and compare the histomorphological, immunohistochemical, and functional alterations resulting from perineural or intraperitoneal ozone therapy in an experimental rat model after a crush injury of the sciatic nerve. The present study demonstrated that both ozone treatment groups exhibited efficacy with respect to myelin degeneration, vascular wall thickness, and toluidine blue staining, with the intraperitoneal ozone treatment being more effective than the perineural treatment compared to the control group. Both perineural and intraperitoneal ozone treatments were similarly effective in vascular congestion, inflammation, and apoptosis. Notwithstanding, only the perineural ozone treatment exhibited the most pronounced improvement in Schwann cell proliferation.

The healing process following a nerve crush injury is significantly hindered by an elevation in free radical production resulting from reperfusion, rather than the inflammatory response and edema that typically accompany neuroinflammation. The free radicals cause the lipid peroxidation of neurovascular cells and lead to oxidative stress. For this reason, the potential of antioxidant therapies to facilitate peripheral nerve recovery has been investigated [5,18]. Given that the antioxidant system is not stimulated by ozone therapy when the concentration is below 20 μg/mL, and that pain is reported during application at concentrations above 50 μg/mL, the therapeutic ozone concentration is maintained at 20–50 μg/mL [6,19]. In this context, the same concentration range was also used in studies investigating the effects of the treatment in peripheral nerve injury models [5,7,13]. In the present study, the selected concentration was 30 μg/mL, which is within the mild level (30–50 μg/mL), for the purpose of stimulating the antioxidant system.

In a previous study utilizing a rat model with experimental sciatic crush injury, the efficacy of ozone therapy was evaluated in comparison with methylprednisolone (MP). The first group received intraperitoneal ozone at a concentration of 20 mcg/mL, the second group received 2 mg/kg MP, the third group received a combination of ozone and MP, and the fourth group received saline alone. A 14-day treatment period was applied to all groups. The combined treatment was reported to cause lower degeneration and inflammation and higher perineural vascular proliferation in peripheral tissues [13]. Somay et al. observed a decrease in vacuolization, vascular congestion, edema, hemorrhage, and fibrosis following the rectal administration of systemic ozone therapy at a dose of 0.7 mg/kg for 10 days [14]. In a separate study employing a facial nerve injury model, the researchers observed a reduction in vascular congestion and macrovacuolation in the group that underwent 1.1 mg/kg intraperitoneal ozone therapy for a 30-day period [5]. Similarly, in our study, myelin degeneration, vascular wall thickness, vascular congestion, and inflammation improved histomorphologically in both ozone treatment groups.

The TUNEL staining results of our study indicated that both types of ozone treatment resulted in a significant reduction in nerve apoptosis. Although gene expressions were not investigated in our study, Güçlü et al. proposed that the potential mechanisms for the protective effect of ozone therapy may be associated with reduced gene expression levels of caspase-1, 3, and 9, along with cell apoptosis-related gene expression [20].

A study was conducted by Lin et al. to investigate the effect of perineural ozone at varying concentrations (10, 30, 50, and 80 µg/mL) on the intact sciatic nerve in rats. The findings indicated that the concentrations of 10, 30, and 50 µg/mL can be safely utilized without compromising the structural integrity of the sciatic nerve [6]. Nevertheless, to the best of our knowledge, only one study conducted a sciatic nerve injury model and applied a perineural ozone injection. The present study demonstrated that a single application of 3.75, 7.5, or 15 µg/0.5 mL of ozone markedly reduced neuropathic pain mediators, including mechanical allodynia and thermal hyperalgesia [12]. This study is the first to determine the therapeutic effect of perineural ozone therapy on sciatic crush injury using histomorphological and immunohistochemical methods. The potential benefits of perineural ozone therapy may be attributed to the increase in superoxide dismutase (SOD) the production and reduction in oxidizing reagents (ROS), as well as the normalization of cytokine and prostanglandin levels, which exhibit anti-inflammatory and analgesic properties similar to those observed in the intraforaminal administration of ozone for nerve root compression in disc herniations [21].

Schwann cell proliferation constitutes an initial nerve response following injury [14,22]. In the study by Somay et al., S100 protein immunoreactivity, which indicates Schwann myelin sheath thickness, was found to be significantly greater in rats with sciatic crush injury treated with rectal ozone compared to the controls [14]. In contrast, the present study revealed that only rats treated with perineural ozone therapy exhibited a notable enhancement in Schwann cell proliferation. In a study by Ogut et al., a more severe nerve injury model was created with a sciatic incision. Rats were administered 35–40 µg/mL intraperitoneally every day for two months, resulting in a significant increase in Schwann cells. In addition, in this study, significant sensory recovery occurred in rats at the 2nd week postoperatively, while motor recovery was observed after the 4th week [7]. Similarly, our study demonstrated an improvement in both ozone treatment groups with regard to sensory function as evaluated at 15 days post-crush injury. The lack of a significant improvement the groups in the rotarod test may be attributed to the fact that motor functions began to improve after the 30th day.

Clinical methods such as nerve conduction studies and electromyography are also available for the functional evaluation of peripheral nerves [11], but imaging methods such as diffusion tensor imaging are also used because they are invasive and provide limited information. Diffusion tensor imaging (DTI), a magnetic resonance imaging technique that measures the effects of membranes on water molecule diffusion, can distinguish between healthy, transected, and regenerating nerves [23]. DTI has also been used in the literature to determine the severity of nerve injury in rats by its ability to differentiate between different degrees of partial nerve transections [24]. In addition, sciatic nerve fascicle differentiation and insights into nerve microanatomy can be assessed with high-resolution ultrasound (HRUS), a non-invasive method. Snoj et al. used HRUS, which can accurately depict small nerve fascicles, to evaluate the sciatic nerve in a human cadaver [25].

The fact that these imaging methods were not used as evaluation methods in our study is one of the limitations. The other limitations of our study are the short follow-up period after the treatments and the absence of electrophysiological, biochemical, and electron microscope evaluations. Another limitation is that the histological scoring system is a subjective method, even though it was evaluated by an experienced expert in the field. However, further studies are needed to confirm the benefits of ozone therapy in human nerve injury and to elucidate the optimal treatment dose range, duration, and mechanisms of ozone.

## 5. Conclusions

The present study demonstrates that perineural and intraperitoneal ozone treatments accelerate nerve regeneration, resulting in histopathologic improvement, a reduction in inflammation and apoptosis, and an enhancement in nerve function in rats with a sciatic nerve crush injury. Additionally, this study is important in demonstrating the efficacy of perineural ozone therapy as an alternative to systemic ozone treatments in nerve injury therapy. These findings suggest that ozone therapy may be a promising treatment option for nerve injuries in humans, although further research is needed to determine the optimal dosing and administration protocols.

## Figures and Tables

**Table 1:** Comparison of the functional assessment data within and between groups.

Variables	Control Group (n = 8)	Paralyzed Group (n = 10)	Perineural Ozone Group (n = 10)	Intraperitoneal Ozone Group (n = 10)	*p* Value	Post hoc Dunn’s Test
Pinch test score (pre-treatment)	3 (3–3)	0 (0–1.25)	0 (0–0)	0 (0–1)	<0.001	1–2, 1–3, 1–4
Pinch test score (post-treatment)	3 (3–3)	0 (0–1.25)	2.5 (1.75–3)	2.5 (2–3)	0.001	1–2, 2–3, 2–4
*p* * value	0.607	0.867	<0.001	0.003		
Rotarod	12.5 (5–30.25)	7 (0–11.5)	8 (0–15.5)	9 (5.25–13.5)	0.402	NS

Median (25–75%); NS: not significant; *p* *: statistical difference between before and after treatment within the group.

**Table 2:** Comparison of microscopy analysis results between groups.

	Control Group (n = 8)	Paralyzed Group (n = 10)	Perineural Ozone Group (n = 10)	İntraperitoneal Ozone Group (n = 10)	*p*-Value	Post hoc Dunn’s Test
Myelin Degeneration	0 (0–0)	3 (2.75–3)	1 (0–1)	0.5 (0–1)	<0.001	1–2, 1–3, 2–3, 2–4
Vascular Congestion	0 (0–0.75)	3 (2–3)	1 (0–1)	1 (0–1)	<0.001	1–2, 2–3, 2–4
Vascular Wall Thickness	0 (0–0)	3 (3–3)	1 (1–1.25)	0 (0–0.25)	<0.001	1–2, 1–3, 2–3, 2–4, 3–4
Inflammation	0 (0–0)	2 (1–2)	0 (0–1)	0 (0–0.25)	<0.001	1–2, 2–3, 2–4
Schwann Cell Proliferation	0 (0–0)	1 (1–1.25)	2 (2–3)	1 (0–1)	<0.001	1–2, 1–3, 2–3, 3–4
Toluidine Blue	0.5 (0–1)	2 (2–3)	1.5 (1–2)	1 (1–2)	<0.001	1–2, 1–3, 2–3, 2–4
TUNEL	0 (0–0)	3 (2–3)	1.5 (1–2)	1 (1–1.25)	<0.001	1–2, 1–3, 1–4, 2–3, 2–4

Median (25–75%).

## Data Availability

The original contributions presented in this study are included in the article. Further inquiries can be directed to the corresponding author.
